# Exploring the impact of the national extended access scheme on patient experience of and satisfaction with general practice: an observational study using the English GP Patient Survey

**DOI:** 10.3399/BJGPO.2022.0013

**Published:** 2022-05-18

**Authors:** Patrick Burch, William Whittaker

**Affiliations:** 1 National Institute for Health Research School for Primary Care Research, Centre for Primary Care, Institute of Population Health, University of Manchester, Manchester, UK; 2 Manchester Centre for Health Economics, University of Manchester, Manchester, UK

**Keywords:** large database research, practice organisation, continuity of patient care, patient satisfaction, general practice

## Abstract

**Background:**

Extended access services were introduced to help stop declining patient satisfaction with access to general practice. There has been no evaluation, at a practice population level, as to how the introduction of these services has impacted patients.

**Aim:**

To explore the association between practices offering extended access and patient responses to the GP Patient Survey (GPPS).

**Design & setting:**

An observational study was carried out. Patient experience data were taken from the national GPPS in England (2018 and 2019). Data on the provision of extended access services were sourced from NHS England. The analyses considered potential confounding factors. These were sourced from publicly available data about practice characteristics from NHS Digital, NHS England, and government websites.

**Method:**

The percentage of patients reporting positive responses to questions related to satisfaction with access, continuity of care, and overall satisfaction were modelled. The association between these outcomes and the provision of extended access were estimated via multivariable fixed-effects linear regression.

**Results:**

There were no associations between practices offering extended access services and key indicators of patient experience or satisfaction at a practice population level.

**Conclusion:**

Extended access has a cost of an estimated 250 million GBP per year. While there is a body of work that finds associations with emergency department use reduction, at a practice population level, in this study it has been found that extended access had no measurable impact. This may be because extended access services are only used by a small number of patients, and its introduction has not significantly impacted general practices and most general practice patients.

## How this fits in

The national extended access scheme was introduced between 2015 and 2018 with the purpose of improving patients’ experiences of accessing general practice. Critics of the scheme were concerned that it may have negative impacts, including on continuity of care. This study shows that, at a practice level, the scheme has had no measurable impact on patient experience of access, continuity of care, or overall satisfaction with general practice.

## Introduction

Recent government policy has prioritised providing patient access to routine GP services in the evenings and at weekends. The reason for this, given by NHS England, was that public satisfaction with access, as measured by the GPPS, had been declining.^1^ In 2016 plans were announced for all clinical commissioning groups (CCGs) in England to commission and fund extended access services. By 2017–2018, half of England had extended access appointments in the evenings and/or weekends.^2^ In February 2018 it was announced that the entire English population would have extended access appointments by October 2018.^2^

It was not mandated as to how practices should deliver their extended access. National data and local service evaluations have suggested that, although many practices do offer some of the extended hours from within the practice premises, a ’hub and spoke’ model of delivering extended access appointments appears to have become the default in many areas.^3–6^ These ‘extended access hubs‘ provide a service from a single location, offering appointments to patients from several different local practices. Although practices have previously worked together to provide urgent care services, the current extended access policy has led to the creation of routine bookable general practice appointments that are often located away from a patient’s regular practice, with another provider. The movement of routine care away from a practice may reduce continuity of care, a marker of healthcare quality associated with several positive health-related outcomes.^7–9^ However, by creating additional appointments, extended access may relieve some of the pressure on practices and could potentially improve patient satisfaction with access and with general practice overall.

Analysis of extended access has focused on the number of appointments being offered, the demographic features of those attending, or the impact that extended access has had on emergency department use.^4–10–12–12^ There is little work that has looked at how extended access services may have affected users of general practice, beyond the confines of a single service. The service may impact on patient experience of those directly using extended access and also indirectly on those not using it (for example, by reducing pressures for core hour services).

In this article, GPPS data are used to examine, at a practice level, whether there is an association between the introduction of widespread extended access services and the following: 1) patient ability to obtain relational continuity (see the same doctor when they want to); 2) patient satisfaction with access; and 3) overall satisfaction with general practice. It is hypothesised that an increase in extended access days offered will cause an increase in patient satisfaction with access but a drop in continuity.

## Method

Multivariable linear regression models are estimated to assess associations of continuity and satisfaction measures with the number of extended access days offered by a practice. The authors control for a range of practice characteristics that may confound associations.

### Extended access data

Data on the number of days and type of extended access offered from all practices in England were collected biannually by NHS England from October 2016 to September 2018. Data include each weekday of extended access offered both from within the practice and in collaboration with another organisation. For the purposes of this study, appointments offered in collaboration with other practices have been referred to as external appointments. Data were compiled from responses by practice staff to whether practices offered extended access appointments ‘at your practice‘ or ‘through a group‘. These questions were open to interpretation by the person answering them. It is possible that group appointments (that is, external provision of extended access), which are hosted at a practice, have been recorded as internal practice provision of extended access. From 1 October 2018 onwards, all CCGs were obliged to be offering their patients extended access appointments at evenings and weekends. Data to confirm to what extent this took place was not available.

### Measures of patient experience and satisfaction

Data on patient experience were sourced from the GPPS, these included measures of access and continuity. The GPPS is an annual survey sent out in January of each year to around 2.5 million people. The survey underwent a redesign for 2018, making it unreliable to compare data from 2018 with earlier years.^13^ Patients surveyed are aged ≥16 years and have been registered at their general practice for at least 6 months. The response rate in 2018 was 34%.^14^ Results from the survey are weighted to make the survey responders representative of the practices’ patient demographic characteristics.

Questions relating to longitudinal continuity, satisfaction with access, and overall experience were chosen for analysis. These were obtained from the following survey questions:

- **How often do you see or speak to your preferred GP when you would like to?** This was asked to those patients with a preferred GP. Variable: percentage for each practice answering ‘Always or almost always‘ or ‘A lot of the time‘.
- **How satisfied are you with the general practice appointment times that are available to you?** Variable: percentage for each practice answering ‘Very satisfied‘ or ‘Fairly satisfied‘.
- **Overall, how would you describe your experience of your GP practice?** Variable: percentage for each practice answering ‘Very good’ or ‘Fairly good’.

**Figure 1.**
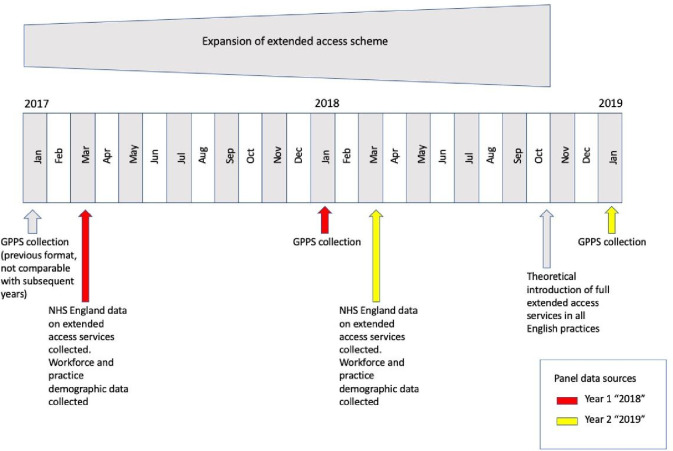
Timeline showing data collection points. GPPS = GP Patient Survey.

For the outcome variables examined, the GPPS asks patients to consider their response at the time of filling in the survey. Answers to the GPPS are likely to reflect an accumulated experience with general practice over time, with several questions specifically asking patients to consider their experience over the previous 12 months. As illustrated in Figure 1, GPPS data collected in January 2018 and January 2019 were used. The GPPS results were paired with extended access data from March of the previous year (for example, January 2018 GPPS results are combined with extended access data from March 2017). This approach assumed no change in the provision of extended access between March and the survey completion date the following January. This assumption may undercount the provision of extended access as practices move towards meeting the national requirements for extended access provision. An alternative approach would be to use the October reported measure of extended access; however, this may overstate provision of extended access over the previous 12 months.

### Potential confounding variables

Factors that have previously been shown to be associated with continuity and access were examined when building the models.^15–17^ These factors were included to reduce potential confounding on the extended access measures should they be associated with practice adoption of extended access. The factors included the number of patients registered at the practice, number of GPs per thousand patients, total number of GPs per full-time equivalent (FTE) GPs, percentage of patients aged <5 years, percentage of patients aged >75 years, percentage of male patients, number of patients with long-term conditions, and type of practice contract. While FTE nurses per thousand patients and patient ethnic group composition have been found to be associated with continuity and access, data for these measures were not available for all practices. All data used were publicly available and extracted from the GPPS or from NHS Digital, NHS England, or other UK government websites.^18–20^

### Statistical analysis

Stepwise Ordinary Least Squares (linear) regression models were estimated with standard errors robust to heteroscedasticity. The regressions included practice dummies (fixed effects) to remove any time invariant unobservable differences between practices that may confound the association between measures of continuity and extended access. Stata (version 17) was used for analysis.

### Sensitivity analysis

For each outcome measure, an additional model was constructed that included the number of nurses in the practice per 1000 patients and patients’ ethnic group. This was not included in the primary analyses as complete data were not available for practices. A total of 4706 (81%) practices had complete data. These variables were added into the main models and run on this smaller sample.

A separate linear regression using a dummy binary variable (instead of the number of extended access days offered) to represent the absence of extended access was estimated.

Additional sensitivity analyses sought to assess whether the location of extended access offered by the practice influenced the results. Three dummy variables were created, reflecting whether practices offered within practice extended access appointments and/or had access to extended access via group or external appointments.

## Results

A total of 5829 practices (from a total of 7213 in England) were included in the sampl (see Supplementary Figure S1). These practices contained complete data for the measures used in the GPPS (Figure 1). Practices included in the analysis were slightly larger (average 8775 versus 8356 patients) but otherwise similar when compared with all practices (see Supplementary Table S1).

### GPPS responses

Table 1 shows the average values for each of the outcome measures in the analyses. There was a drop in all markers of practice rates of continuity, access, and satisfaction between 2018 and 2019. The average values for all explanatory variables used in the analyses are provided in Table 2.

**Table 1.** Mean values for outcome variables for all practices included in the model (*n* = 5829)

Question and variables	Mean 2018, %	Mean 2019, %	Difference, %
**How often do you see or speak to your preferred GP when you would like to?^a^** ‘Always or almost always‘ or ‘A lot of the time‘	51.8	49.6	–2.2^b^
**How satisfied are you with the general practice appointment times that are available to you?** ‘Very satisfied‘ or ‘Fairly satisfied‘	67.6	66.5	–1.1^b^
**Overall, how would you describe your experience of your GP practice?** ‘Very good’ or ‘Fairly good‘	84.6	83.9	–0.7^b^

^a^This was asked to those patients with a preferred GP. ^b^ *P*<0.05

**Table 2.** Source and average values for explanatory variables used in the analyses

Category	Source	Mean 2018	Mean 2019	Combined means
Extended access days offered, n	NHS England – practice submitted data	3.46	4.41	3.94
Patients registered with practice, n	GPPS	8909	8640	8775
Male patients registered with practice, %	GPPS	49.2	49.2	49.2
Prevalence of long-term conditions, %	GPPS	51.3	52.4	51.8
FTE GPs per 1000 patients, n	NHS Digital and GPPS	0.54	0.50	0.52
GPs per FTE GPs, n	NHS Digital	1.38	1.39	1.38
Patients aged <5 years, %	NHS Digital	5.5	5.6	5.6
Percentage of patients aged >75 years, %	NHS Digital	8.0	7.8	7.9

FTQ = full-time equivalent. GPPS = GP Patient Survey.

### Extended access data

**Figure 2.**
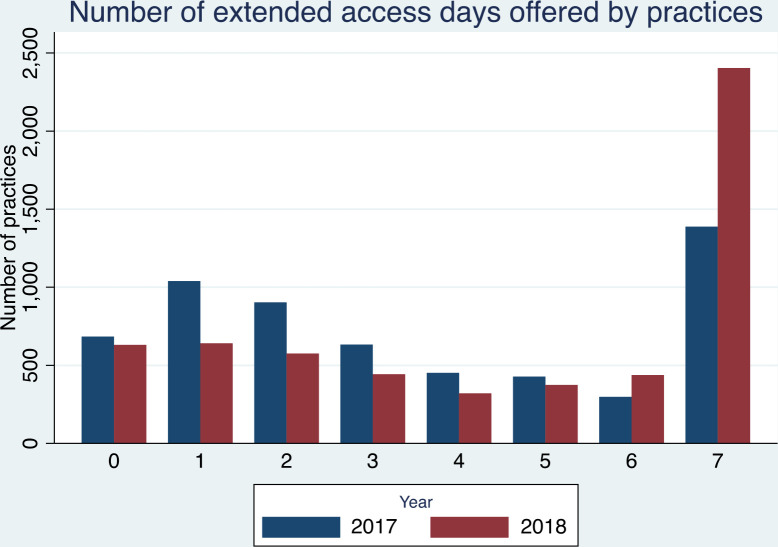
Number of extended access days offered by practices

Figure 2 shows the number of extended access days offered by practices (mean 2017, *n* = 3.5; mean 2018, *n* = 4.4).

Table 3 shows the location of extended access services offered by practices. Between 2017 and 2018 there was a 6% decrease in the number of practices offering internal extended access appointments (2017, *n* = 4766; 2018, *n* = 4494). During the same period, the number of practices offering some form of external extended access service rose by 59% (2017, *n* = 2050; 2018, *n* = 3257).

**Table 3.** Type or location of external access offered by practices

Category	March 2017, % (*n*)	March 2018, % (*n*)
Practices with no extended access	11.7 (684)	10.8 (631)
Practices with only internal extended access	53.0 (3091)	33.3 (1941)
Practices with only external extended access	6.4 (375)	12.1 (704)
Practices with both internal and external extended access	28.7 (1675)	43.8 (2553)

### The effect of extended access

Table 4 presents the estimates from the regression analyses for each of the practice measures. There were no associations found between the number of extended access days provided by a practice and any of the three patient scores reported in the GPPS. Practice size was significantly correlated with poorer results in all three domains of practice measures.

**Table 4.** Regression outputs. *N* = 5829 practices. Estimates are from linear regression models and include practice fixed effects (not reported), standard errors are robust to heteroscedasticity.

Category	Percentage of patients satisfied with access	Percentage of patients able to speak to preferred GP	Percentage of patients satisfied with overall experience
	Beta (95% CI)	Beta (95% CI)	Beta (95% CI)
Number of extended access days	0.0002(–0.0008 to 0.001)	0.00003(–0.001 to 0.001)	–0.0006(–0.001 to –0.0001)
Percentage of male patients	–0.17(–0.051 to 0.16)	0.046^a^(0.002 to 0.90)	–0.020(–0.046 to 0.007)
Number of thousand patients	–0.002^a^(–0.005 to –0.002)	–0.003^a^(–0.005 to –0.0008)	–0.003^a^(–0.004 to –0.0009)
FTE GPs per thousand patients	0.006(–0.011 to 0.023)	0.018(–0.002 to 0.038)	0.012(–0.0003 to 0.024)
Percentage of patients aged <5 years	0.007(–0.001 to 0.016)	0.008(–0.004 to 0.020)	0.0036(–0.003 to 0.010)
Percentage of patients aged >75 years	–0.002(–0.011 to 0.006)	–0.0004(–0.001 to 0.011)	–0.002(–0.008 to 0.005)
Percentage of patients with long-term conditions	0.023(–0.006 to 0.052)	0.056^a^(0.017 to 0.095)	0.010(–0.013 to 0.032)
Year (2019)	–0.012^a^(–0.015 to –0.009)	–0.022^a^(–0.026 to –0.018)	–0.006^a^(–0.009 to 0.004)

*^a^P*<0.05. FTE = full-time equivalent.

A range of sensitivity analyses were conducted and are reproduced in the supplementary material. The analyses were replicated with the addition of the number of nurses in the practice per 1000 patients and patients’ ethnic group (see Supplementary Table S2). Neither variable was found to be significantly correlated with any of the dependent variables and their addition, in this subset of practices, did not alter the results of the original models significantly. A model using the presence or absence of any extended access service (rather than the amount offered) showed no significant association between the offering of extended access and the GPPS results (see Supplementary Table S3). An alternative model included whether the practice offered no extended access, external extended access, within practice extended access, or a mixture of the two. In this model the presence or location or form of extended access had no significant association with GPPS results (see Supplementary Table S4).

## Discussion

### Summary

This study sought to assess whether there were associations between the provision of extended access and patient measures related to access, ability to see preferred GP, and overall satisfaction with general practice, at a practice population level. No evidence was found of a statistically significant association between extended access provision and any of the practice measures.

### Strengths and limitations

To the authors’ knowledge, this is the first study that has used national data on practice extended access hours to quantify the impact of the introduction of the extended access scheme on patient-reported measures. Although the scheme may benefit patients who utilise extended access appointments, this study aimed to assess the impact of the scheme on practice-level rates of satisfaction (the primary motivation of the policy). The study utilised data from a period when there were considerable differences in the provision of extended access between practices before the introduction of nation-wide coverage. The use of fixed-effects modelling eliminates time invariant confounding factors that may bias cross-sectional models.

The GPPS is the largest validated survey of patient-reported measures that exists in England. However, it is completed by just over 1% of English general practice patients. It is likely that the GPPS is too insensitive an instrument to pick up any impacts caused by a service used by only a small number of patients. Nonetheless, extended access may indirectly impact on patients not using the service by impacting on pressures to routine general practice. Further, the aim of the service was largely motivated by these measures and a desire to improve them.^1^

Practice-level GPPS data are not an appropriate instrument for evaluating individual-level patient satisfaction with extended access services, and this study did not attempt to do this. The aim was to test the hypothesis that practices with access to extended access have patients who report better access, continuity, and satisfaction on the GPPS. Practice-level assessment did not support stratification by subgroups to explore whether there were impacts on groups of patients. It is possible that when analysed separately, subgroups of patients may differ significantly in the association that extended access has with their GPPS scores. The extended access appointment data have limitations. It was not possible to know the location of appointments or whether they were utilised. The GPPS questions on access and continuity only capture one aspect of these issues and ignores their complex multifaceted nature.^21,22^

### Comparison with existing literature

A longitudinal study of 2013–2018 GPPS data into the effects of practices collaborating closely with others found no significant association between practice collaboration and patient experienced continuity.^16^

A cross-sectional study using 2013–2014 GPPS data grouped practices into those offering extended access and those not.^23^ This found a positive association between patient satisfaction with access and scheme participation. The study found an improvement of around 1% in satisfaction with access for those practices that participated in extended access. They were unable to examine any dose effect. The extended access scheme they examined was different to that examined here in terms of times, locations of appointments, and funding arrangements. Extended access, in the context of this older study, was a non-compulsory scheme where some practices offered early morning, evening, or weekend appointments to their existing patients. The difference in schemes and methods of analysis makes direct comparison of results problematic.

### Implications for research and practice

NHS England states that extended access services must provide an addition of 45 minutes of consultations per 1000 patients per week. In an average-sized practice (8356 patients), this equates to around 6 hours additional appointments per week. However, despite offering comparatively few appointments, extended access has generated additional complexity within general practice systems.

The political imperative to ‘improve access‘ for working adults to general practice was the driving force behind the introduction of a universal extended access policy. However, the concept of access encompasses more than just being able to book an appointment at the earliest or most convenient time.^24^ There is an opportunity cost behind all health service initiatives. NHS spending on providing extended access general practice services in 2018–2019 was likely to have been in the region of 250 million GBP.^25^ While extended access may benefit certain demographics, this study has failed to show any wider impact on the general patient population. There is evidence from service evaluations that some external extended access appointments had not been filled, were being dominated by patients from a particular practice, or were being used simply because there were not enough day-time appointments.^4–11,26,26^ Although localised to one area, a survey of 1600 patients using extended access in the Salford area in 2017 to 2019 found that over half of patients were using extended access services because they could not get an appointment at their own general practices, not because they found the appointment or location times convenient.^5^ With the increased availability of telephone and video consulting following the COVID-19 pandemic, it is questionable whether there is now as much demand for access to appointments outside of the regular working day.

Subanalysis of individual patient groups may shed light on whether groups benefit from extended access. More research is needed into how the COVID-19 pandemic has impacted on the need for extended access appointments. Work is also required to see if patients who are seen outside of their regular practice are treated differently to those seen within their practice, and how their treatment and follow-up is integrated into the wider primary care system.
